# Airborne Tire Wear Particles: A Critical Reanalysis of the Literature Reveals Emission Factors Lower than Expected

**DOI:** 10.1021/acs.estlett.4c00792

**Published:** 2024-11-24

**Authors:** Siriel Saladin, Adam Boies, Chiara Giorio

**Affiliations:** † Yusuf Hamied Department of Chemistry, University of Cambridge, Cambridge CB2 1EW, United Kingdom; ‡ Department of Engineering, University of Cambridge, Cambridge CB2 1PZ, United Kingdom

**Keywords:** nonexhaust emissions, microplastics, tire abrasion, tire debris, particulate matter, PM_10_, TWP, TRWP

## Abstract

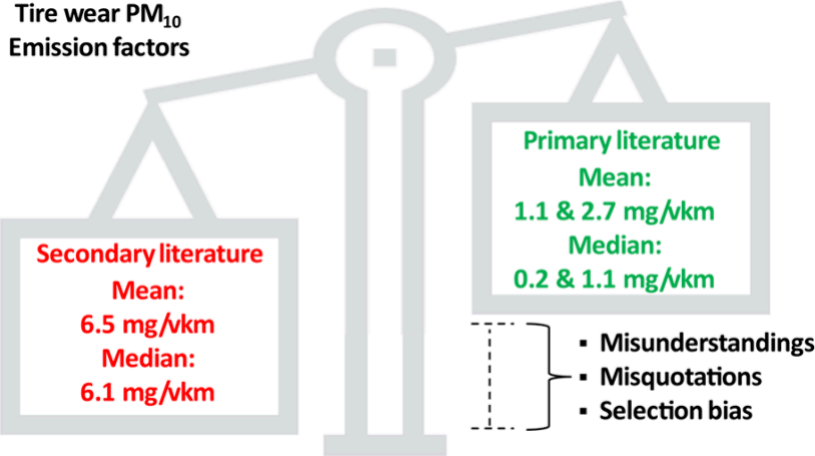

Tires are a ubiquitous part of on-road transport systems serving as the critical connecting component at the interface of the motive power and road surface. While tires are essential to automobile function, the wear of tires as a source of particulate air pollution is still poorly understood. The variety of reported emissions found in the secondary literature motivated us to summarize all known mass-based tire wear emission factors for light-duty vehicles in primary research. When excluding road wear and resuspension, mean emissions of 1.1 mg/km/vehicle (median 0.2 mg/km/vehicle) were found for tire wear PM_10_ and mean emissions of 2.7 mg/km/vehicle (median 1.1 mg/km/vehicle) when including studies with resuspended tire wear. Notably, these factors are substantially lower than broadly cited and accepted factors in the secondary literature with mean emissions of 6.5 mg/km/vehicle (median 6.1 mg/km/vehicle). As revealed by our analysis, secondary literature reports emission factors systematically higher than those of the primary sources on which they are based. This divergence is due to misunderstandings and misquotations that have been prevalent since the year 1995. Currently accepted mass-based emission factors for directly emitted airborne tire wear particles need revision, including those from the United States Environmental Protection Agency and the European Environment Agency.

## 1 Introduction

The World Health Organization estimated a global mortality of 4.2 million premature deaths due to outdoor air pollution for the year 2019.^1^ This number related to particulate matter matches the estimated mortality of dementia^2^ (1.62 million including Alzheimer’s disease), road traffic injuries^3^ (1.19 million), suicides^4^ (700,000), and malaria^5^ (608,000) combined, prompting governments to regulate particulate matter with aerodynamic diameters smaller than 10 μm (PM_10_) and 2.5 μm (PM_2.5_). Road transport is reported to account for 11% of the total PM_10_ primary emissions in the European Union, with tire wear as a relevant source.^6^ Tire wear emissions are expected to increase because of a persistent trend toward heavier vehicles in conjunction with transport electrification.^7,8^ As a result, the European Commission announced the intention to regulate tire wear emissions as part of the upcoming Euro 7 standards, which will be the first emission standard worldwide to move beyond regulating tailpipe emissions.^9^

The prevalence of tires and lack of alternative technologies motivates the study of tire wear as an aerosol emission source and eventually as a health risk, given that toxicological effects have been observed for tire-related airborne particles.^10−12^ Our own work has sought to measure tire elemental tracers for source apportionment and quantification of tire particles,^13^ which are reported to have sizes ranging from less than 10 nm to more than 100 μm.^14,15^ The emission factors of airborne tire wear particles must be estimated such that meaningful health impacts can be studied based on representative exposures. Further, these factors play a critical role in the development of standards for industrial manufacturers and whether environmental policies should be developed targeting tire emissions, such as PM_10_. The absolute magnitude of mass- and eventually number-based airborne emissions from tires needs to be assessed and critically reviewed by academia, industry, and policymakers.

The currently reported emission factors for tire wear PM_10_ from light-duty vehicles range over a span of 5 orders of magnitude: from 0.00027 to 44 mg/vkm, expressing the mass of emitted tire wear per vehicle-kilometer.^16,17^ For context, the European Union^18^ and the United States^19^ currently regulate exhaust PM from cars to 4.5 mg/vkm and 1.9 mg/vkm, respectively. In other words, one end of the reported range indicates that the mass of tire wear PM_10_ is relatively insignificant, whereas the other end exceeds the limits for exhaust emissions 10-fold. If there is no consensus, what do the tire wear PM_10_ emission factors of 6.4 mg/vkm from the European Environmental Agency^20^ (EEA) or 5.3 mg/vkm from the United States Environmental Protection Agency^21,22^ (EPA) signify? Motivated by this question, we summarized emission factors reported in the primary literature and compared them with emission factors from scientific reviews, reports, textbooks, and emission inventories. We sought to use the findings to assess the contribution of tire wear to air pollution and discuss a potential discrepancy between measured and propagated emission factors.

## 2 Methods and Materials

Search engines (Google, Scopus, Web of Science, and ResearchGate) were used to identify primary studies estimating emission factors of airborne tire wear particles. Additionally, secondary studies summarizing such factors were identified, along with the references cited by these studies. Both spellings “tyre” and “tire” were considered. Research institutes, environmental agencies, local authorities, and authors of key publications were contacted by e-mail or telephone to obtain additional insights in the case of ambiguities or when a study was not accessible. Our team of authors consisted of native English and German speakers, allowing us to read key publications of both languages. Emission factors from studded tires were excluded due to interference from road wear. Similarly, results were excluded that referred to emergency braking or when primary literature authors expressed concern due to interferences from resuspension caused by insufficient cleaning prior to that run.

### 2.1 Definitions

**Figure 1:**
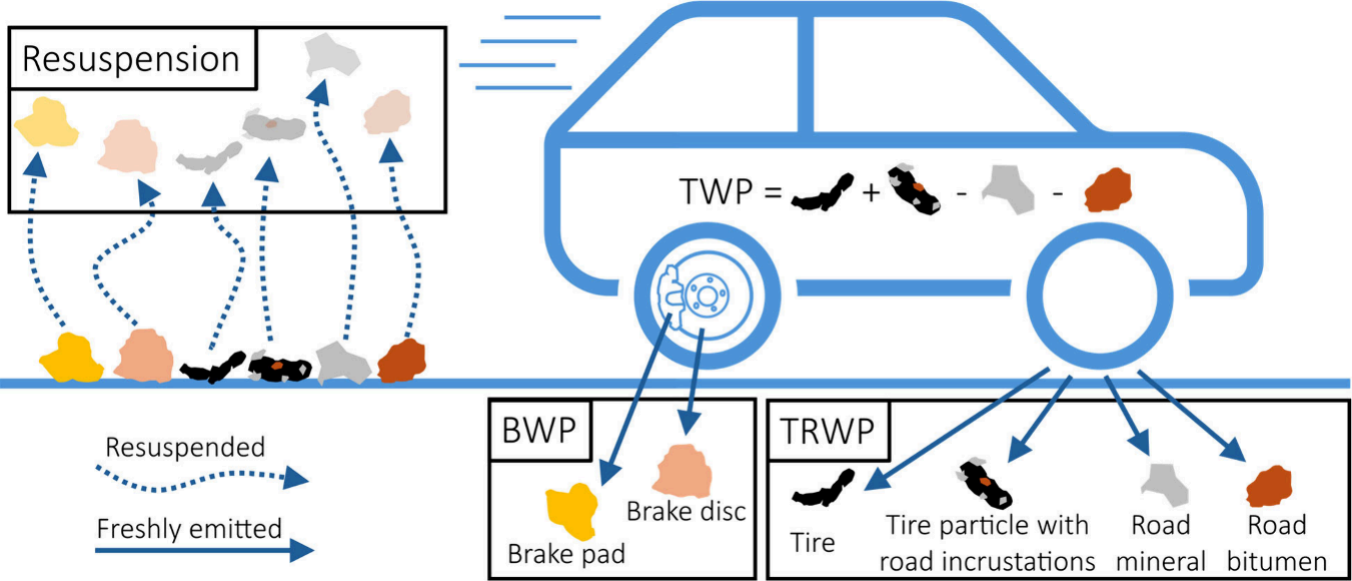
Illustration of our definitions for tire wear particles (TWP), tire and road wear particles (TRWP), brake wear particles (BWP), and “resuspension”. These definitions for TWP, TRWP, and BWP are limited to freshly emitted particles, while any resuspended particles are classified as “resuspension” to avoid double counting.

In this study, we define tire wear PM_10_ or tire wear particles (TWP) as particles directly emitted from tires due to wear. Brake wear particles (BWP) and road wear particles are the equivalents for the brakes and roads, respectively. Our definition of TWP excludes the contribution from road wear particles. The total TWP and road wear particles is defined as tire and road wear particles (TRWP), which represent a mixture of TWP, road wear particles, and TWP incrusted with road wear particles. TWP, BWP, or TRWP may settle and be resuspended by wind or the wake of passing vehicles. We define resuspended particles as “resuspension” and not as TWP, BWP, or TRWP. These definitions (illustrated by Figure 1) avoid double counting and agree with the practice of EEA^20^ and EPA.^21,22^ However, different definitions can be found for tire wear in the literature. For example, Piscitello et al.^23^ defined tire wear particles as tire wear including road wear, in contrast to Baensch-Baltruschat et al.^24^ who excluded road wear. Similarly, Hicks et al.^25^ and Beddows et al.^26^ excluded road wear in their usage of the term “tire wear”, while including both freshly emitted and locally resuspended tire wear particles.

Some studies have quantified the tire contribution within TRWP, in which case we have taken the tire mass fraction as TWP. Similarly, particulate emissions were classified as TWP when a tire on a wear resistant road surrogate like sandpaper was abraded. All emission factors are given as mass per distance (mg/vkm) expressing milligrams of emitted particles per kilometer driven by a vehicle with four tires. If a reference reported emission factors per tire, it was multiplied by 4 to obtain comparable emission factors, assuming identical emissions for all tires. Total suspended particles (TSP) correspond to airborne particles regardless of the size. PM_10_ aerosolization efficiency indicates the mass fraction of tire wear becoming airborne PM_10_.

Relevant primary literature is defined as studies estimating mass-based emission factors for airborne TWP, TWP+BWP, or TRWP based on their own experiments, aimed at reflecting typical driving conditions. A publication was considered secondary literature (referred to as reviews) when elsewhere reported emission factors were quoted, and no independent measurements of airborne tire particles were performed. A publication was classified as a nonrelevant primary study when airborne particles were measured, but no mass-based emission factors for airborne tire wear were reported. A citation was considered to be inaccurate if the quoted information was not contained in the cited reference.

## 3 Results and Discussion

### 3.1 Emission Factors in the Primary Literature

The accuracy and representativeness of emission factors for airborne tire wear depend greatly on the underlying methodology, as well as the conditions that prevailed during the experiment. Different approaches are subject to different limitations and uncertainties, as described in the Supporting Information (Text S1). This section does not provide a best estimate for a representative emission factor of airborne TWP. Instead, it gives an overview of the emission factors proposed within primary research.

A total of 26 primary studies were found that reported 63 mass-based emission factors for airborne TWP, TRWP, or TWP+BWP from light-duty vehicles or mixed fleets. The term “mixed fleet” refers to traffic characterized by a mixture of different vehicles, such as motorcycles and light- or heavy-duty vehicles. Some authors quantified directly emitted tire-related particles, while others further included the resuspension of these particles. In two studies, no emissions of airborne TWP could be quantified.^27,28^ A few studies additionally examined two-wheelers, three-wheelers, or heavy-good vehicles, whose emission factors were excluded for better comparability. Most authors reported emission factors for PM_10_, whereas only one study^29^ quantified PM_2.5_ and not PM_10_. For better comparability, PM_10_ emissions are targeted hereafter. The reported emission factors should be discussed with respect to the individual limitations and uncertainties of the used approaches. Such an evaluation requires the primary literature to be presented in a comparable yet differentiated manner, which we attempt to provide in Table 1. Some factors were converted by us according to the overview in the Supporting Information.

**Table 1:** Emission Factors in Primary Literature for Airborne TWP, TRWP, and TWP+BWP of Light-Duty Vehicles with Unstudded Tires if Not Otherwise Stated

reference	sampling	speciation	emission (mg/vkm)
TWP TSP			
Williams and Cadle^30^	drum		2–5^a^
Pierson and Brachaczek^31^	tunnel	rubber, zinc	2.5^b^
TRWP PM_10_			
Kupiainen et al.^32^	asphalt track		9 and 11
Gustafsson et al.^33^	asphalt track		0.2–7
Beji et al.^34^	on-road		5.0^c^
Gehrig et al.^35^	asphalt track		0–3^d^^,^^e^
Charbouillot et al.^36^	on-road	density separation	2.64^f^
Alves et al.^37^	asphalt track		2
Khardi^38^	on-road		1.45^c^^,^^g^
Aatmeeyata et al.^39^	concrete drum		0.0037^c^^,^^d^^,^^e^
TWP+BWP PM_10_			
Farahani et al.^40^	roadside	CMB	10.63^g^
Luhana et al.^41^	tunnel	CMB	6.9
TWP PM_10_ (including resuspended TWP)			
Hicks et al.^25^	roadside	zinc	3.5–11.0^b^^,^^g^
Beddows et al.^26^	roadside	zinc, CMB	9.92,^b^^,^^g^^,^^h^ 10.9^b^^,^^g^^,^^h^
Panko et al.^42^	roadside	vinylcyclohexene, dipentene	2.4^b^^,^^g^
Sjödin et al.^43^	roadside	CMB	2.2^b^^,^^g^
De Oliveira et al.^44^	on-road	vinylcyclohexene, phenylcyclohexene	0.15^g^
Abu-Allaban et al.^27^	roadside	CMB	b, g, i
Bukowiecki et al.^28^	roadside	XRF	b, g, i
TWP PM_10_ (directly emitted)			
Rauterberg-Wulff^45^	tunnel	CMB (carbon)	6.1
Zhang et al.^46^	tunnel	CMB	1.1–4.5^b^^,^^j^
Tonegawa and Sasaki^47^	on-road	styrene	4.1^k^
Woo et al.^15^	safety walk drum		0.46–1.34^e^
Zhang et al.^48^	tungsten carbide drum		1.27
Park et al.^49^	safety walk drum		0.144–0.8908
Gehrig et al.^35^	asphalt track	zinc	≤0.80^d^^,^^e^
Allen et al.^50,51^	tunnel	unidentified tracers	0.120–0.354^b^^,^^h^
Kupiainen et al.^32^	asphalt track	CMB	0.17
Kim and Lee^52^	sandpaper drum		0.0011–0.0210^e^
Aatmeeyata et al.^39^	concrete drum	carbon	0.00093^c^^,^^d^^,^^e^
TWP+BWP PM_2.5_			
Fang et al.^29^	tunnel	CMB	0.03–0.48^b^

a Cited in Cadle and Williams.^53^

b Mixed fleet.

c Assumed particle shape and density for mass conversion.

d Extrapolated load assuming linear relationship.

e Potentially underestimated due to abnormally low loads.

f Tire particles with and without road incrustations.

g Includes resuspended tire-related particles.

h PM_1–10_.

i No tire wear PM_10_ found (unclear limit of detection).

j High uncertainty (up to 270% relative standard deviation).

k Assumed no styrene contribution from road wear.^54^

In summary, the identified primary studies reported 35 TWP PM_10_ emission factors ranging from 0.00093 to 11.0 mg/vkm with a mean of 2.7 mg/vkm and median of 1.1 mg/vkm when including estimates for mixed fleets and resuspended TWP. When excluding the estimates for resuspended TWP, a lower mean of 1.1 mg/vkm and median of 0.2 mg/vkm is obtained. The resuspension of TWP may contribute more to PM_10_ than the direct emissions, considering the differences between studies that excluded or included resuspended TWP (Table 1). The mean and median factors for TRWP PM_10_ from light-duty vehicles were 2.7 and 1.5 mg/vkm, respectively. The mass dominating airborne material resulting from the tire–road interaction may abrade from the roadway rather than from the tire. This hypothesis from Pierson and Brachaczek^31^ in 1974 is supported by the results of various studies that chemically characterized airborne TRWP.^32,33,35,37,39,43,55−57^ When considering elsewhere reported tire tread losses of ∼100 mg/vkm for light-duty vehicles under typical conditions,^17,20^ the available data, including the most conservative estimates, imply tire wear PM_10_ aerosolization efficiencies below 10% or even below 1%.

Although mean and median are suitable metrics to describe typical emission factors within the primary (or secondary) literature, they are less suitable to identify the most accurate emission factor. Note that the means and medians in this section do not reflect individual uncertainties, limitations, and varieties between different conditions, such as speed or load. However, uncertainties, including systematic deviations, are generally not quantified. The identification of the most accurate factor is, therefore, subject to scientific discussions. For our study, we retained all estimates from Table 1. Consequently, our mean and median emission factors are a general description of primary research and not a best estimate of a true emission factor.

The emission factors in Table 1 range over 4 orders of magnitude, which can partly be explained as not all estimates refer to the same emission type. Nevertheless, a substantial variety can still be observed, even within one emission type. For example, considering directly emitted tire wear PM_10_ of light-duty vehicles, estimates ranging from 0.00093 mg/vkm (Aatmeeyata et al.^39^) to 6.1 g/vkm (Rauterberg-Wulff^45^) can be found. The remaining paragraphs in this section provide important methodological details attempting to explain part of the variety.

The lowest TWP PM_10_ emission factors (<0.1 mg/vkm) were reported by Kim and Lee^52^ and Aatmeeyata et al.^39^ It should be borne in mind that both studies used abnormally low lateral loads due to experimental limitations. Similarly, the worst case estimate of 0.80 mg/vkm from Gehrig et al.^35^ was derived using a relatively low load, which corresponds to approximately one-third of a car. Aatmeeyata et al. and Gehrig et al. corrected for the low load assuming a linear relationship between load and tire wear PM_10_ emissions. However, the validity of this assumption is unclear. Generally, tire wear on nonasphalt surfaces like sandpaper (Kim and Lee) or concrete (Aatmeeyata et al.) could be drastically different to asphalt. Additionally, Schläfle et al.^57^ reported the relevance of third-body particles, since a complete lack of dirt between the tire and the road was observed to prevent the release of fine TRWP. It is further unclear whether electric charges impair the collection efficiency of airborne TWP. These considerations could explain low emission factors compared to other studies.

On the other side, the six highest emission factors (ranging from 6.3 to 11.0 mg/vkm) for tire wear PM_10_ were reported by Hicks et al.^25^ and Beddows et al.^26^ This can partly be explained as both studies used a similar methodology based on roadside increments to quantify the total of directly emitted and locally resuspended TWP. Additionally, both studies used zinc as a tracer, assuming that 50% by mass of the detected zinc originated from tire wear. This assumption dates back to the year 1974 when Pierson and Brachaczek^31^ used the same zinc specificity for tire wear TSP. However, the specificity of zinc for PM_10_ could be lower than generally assumed according to the observations from Wang et al.^58^ and the caution urged by Chen et al.^59^ An overestimated zinc specificity could lead to overestimated emission factors. This may explain why Hicks et al. did not observe a decrease of TWP emissions during the reduced traffic volumes associated with the coronavirus pandemic (unlike BWP).

Panko et al.^42^ quantified roadside tire wear PM_10_ including resuspended TWP in Paris (France) using rubber pyrolysis products as tracers. The authors calculated an emission factor of 2.4 mg/vkm using a box model in combination with traffic data from the Ile-de-France region. It would be misleading to describe this region as urban, although its center is urban. The employed model assumes that the measured TWP concentration at the roadside in Paris is representative for Ile-de-France. However, it seems that the TWP concentrations near the emission source are higher than, for example, in forests or on agricultural land,^60^ thus indicating that the emission factor of Panko et al. potentially represents a rather conservative estimate.

The highest estimate (6.1 mg/vkm) for tire wear PM_10_ without resuspension was found in the doctoral thesis from Rauterberg-Wulff^45^ (1998). The author has provided us with a printed copy, since this dissertation, written in German, is unavailable online. Its emission factor is widely quoted and forms the rationale of the current tire wear PM_10_ emission factors from both EPA and EEA (see Text S2). To make it more accessible, we have briefly summarized the underlying methodology in the Supporting Information (Text S3). The methodology is based on a chemical mass balance, assuming no contribution from road wear to carbon in PM_10_. This assumption seems uncertain in view of the considerations in the Supporting Information (Text S3), implying that the estimate of 6.1 mg/vkm is potentially overestimated.

### 3.2 Comparison with Secondary Literature

A total of 14 reviews were identified that have summarized mass-based emission factors for airborne tire wear: seven reviews^16,17,23,24,61−63^ published in peer-reviewed scientific journals, three reports^64−66^ from research institutes, one chapter^67^ of a textbook, one report^68^ from the EPA, one report^69^ affiliated with the European Union, and one Web site^70^ affiliated with the United Nations. In all reviews combined, a total of 135 emission factors for airborne TWP, TRWP, or TWP+BWP were found, including duplicates, as multiple reviews have quoted emission factors from the same sources. The reviews did not differentiate between directly emitted and resuspended tire-related particles. Similarly, they did not differentiate between light-duty vehicles and mixed fleets. For better comparability with primary studies, we excluded emission factors for PM_2.5_ or vehicles other than light-duty vehicles. A table of all considered emission factors is provided in the Supporting Information. Note that the EPA and EEA use emission factors for tire wear PM_10_ from light-duty vehicles to derive factors for other vehicles and PM_2.5_ (Text S2).

Notably, clear definitions for “tire wear” were only found in 3 of 14 reviews, while the others provided no definitions or used inconsistent definitions. In the latter cases, we have classified the emission factors as TWP, since the factors were presented in a context characterized by the word “tire” in combination with the absence of the words “road”, “pavement”, “asphalt”, or “resuspension”. This explains why 129 of the 135 identified emission factors from the secondary literature are listed as TWP PM_10_, whereas only 35 of the 58 PM_10_ emission factors from primary research referred to TWP (Table 2).

**Table 2:** Emission Factors (Light-Duty Vehicles and Mixed Fleets) for PM_10_ from TWP and TRWP According to Primary Literature (prim. lit.) and Secondary Literature (sec. lit)

	TWP PM_10_			TRWP PM_10_	
	prim. lit.^a^	prim. lit.^b^	sec. lit.	prim. lit.^c^	sec. lit.
*N* (studies)	16	21	14	8	14
*N* (factors)	23	35	129	21	2
min. (mg/vkm)	0.00093	0.00093	0	0.0037	2
max. (mg/vkm)	6.1	11.0	44	11	9
mean (mg/vkm)	1.1	2.7	6.5	2.7	5.5
median (mg/vkm)	0.2	1.1	6.1	1.5	5.5

a Direct emissions.

b Includes five studies with resuspended TWP.

c Includes one study with resuspended TRWP.

The emission factors for TWP PM_10_ reported by secondary literature ranged from 0 to 44 mg/vkm with a mean of 6.5 mg/vkm and median of 6.1 mg/vkm. These emission factors are in good agreement with the emission factor of 6.4 mg/vkm from EEA^20^ and 5.3 mg/vkm from EPA^21,22^ for light-duty vehicles. However, the mean from secondary literature is 2 times and the median is 6 times higher than the equivalents from the primary literature including studies with resuspended TWP. When excluding studies with resuspended TWP, the secondary literature reports 6 times higher means and 30 times higher medians than primary research. Similarly, the upper end of the TWP range reported by the reviews is 4 times higher than the highest estimate found within primary research.

A detailed comparison between the primary and secondary literature was performed to assess the underlying reasons behind the discrepancy identified in Table 2. For example, the reviews may have quoted studies that we have missed, or we may have included studies that were excluded by the reviews due to high uncertainties. Similarly, the factors in secondary literature may result from “worst case” emission factors implemented for regulatory purposes. To elucidate these hypotheses, we have assessed the citations from the reviews according to the tire wear definitions in these reviews. The references cited by the reviews were classified as either relevant primary literature, nonrelevant primary literature, secondary literature, or unknown in case the cited reference was not accessible for us (see definitions for the criteria). Additionally, the accuracies of the citations were classified as either accurate, inaccurate, or unknown. Multiple emission factors from one source quoted by one review were counted as one citation. A detailed table of all citations is provided in the Supporting Information, clarifying the classification for all citations and references.

**Figure 2:**
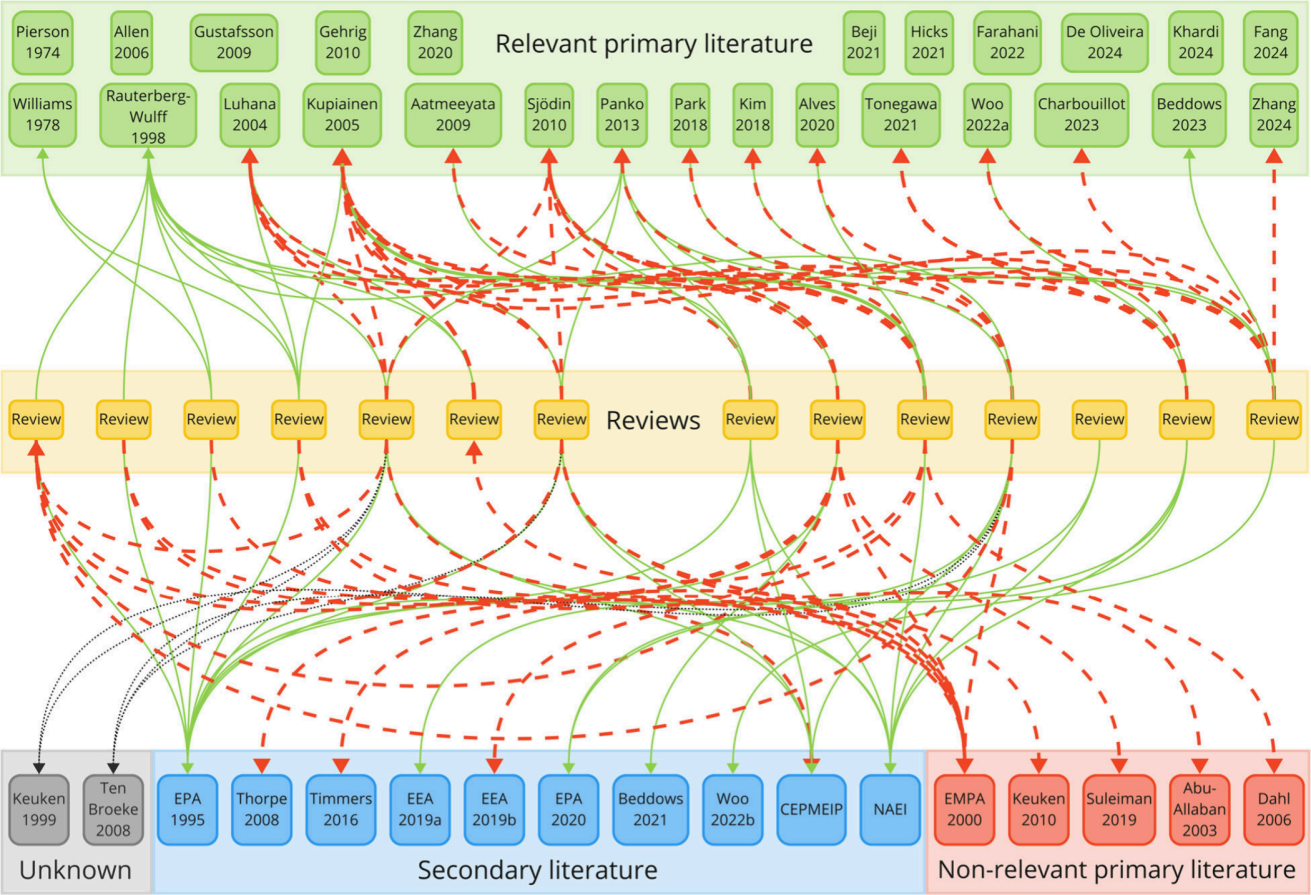
Overview of all 107 citations from 14 reviews to 34 references regarding emission factors for airborne tire wear particles. Accurate and inaccurate citations are highlighted by solid green and dashed red arrows, respectively.

The reviews made a total of 107 citations to 34 different references, whereof we could read all except for 2. The availability of Keuken et al.^71^ is unclear according to correspondence with one of the authors and the issuing research organization. The report of ten Broeke et al.^72^ appears to be secondary literature, albeit written in a language we do not comprehend (Dutch). The reviews did not quote references that we have missed. Figure 2 provides an overview of the analyzed reviews, citations, references, and uncited relevant primary studies.

The reviews referred to relevant primary literature in 56 of 107 citations, while 34 referred to secondary literature, 12 to nonrelevant primary literature, and five citations were unknown. In 13 of 14 cases, the reviews presented an undifferentiated mix of emission factors from primary and secondary research, which may introduce a bias as frequently quoted factors could be given unproportional importance. Many of the cited references like CEPMEIP^73^ or the United Kingdom National Atmospheric Emissions Inventory^74^ (NAEI) are different versions of the same source and are somehow linked to the EMEP/EEA emission inventory guidebook^20^ from the European Union. NAEI quotes the emission factors from the guidebook,^75^ which in turn cites the initially mentioned CEPMEIP database (base year 1995), which in turn was written under the same program as the guidebook. CEPMEIP is not peer-reviewed and does not provide references for TWP. Its developers were unable to explain how the tire emission factors were derived when we contacted them by email. Today’s tire wear emission factors from the EEA and EPA rely on the same rationale from the year 2003 (outlined in Text S2), implying that 19 citations from the reviews to secondary literature are based on two references: EMEP/EEA and CEPMEIP. Both references are linked to each other and were developed 20 years ago. This observation was not mentioned by the 14 reviews.

**Figure 3:**
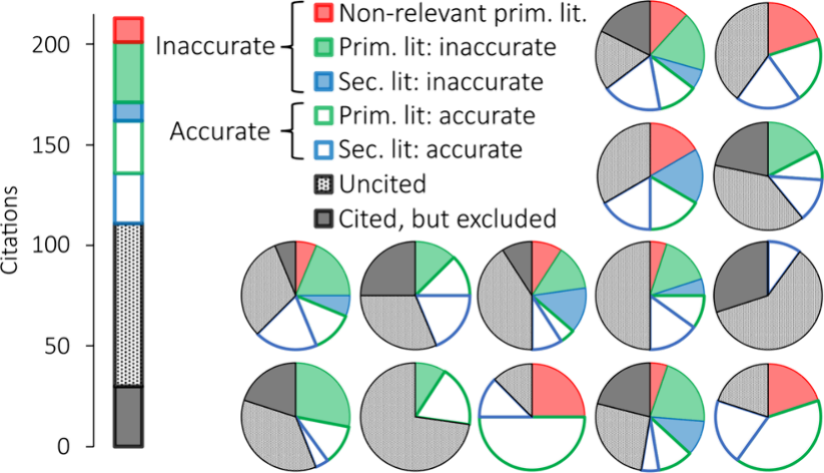
Illustration of the citations from the 14 reviews. Every pie chart represents one review. The bar chart refers to the total of all of the reviews. References that were not considered by the reviews are colored light and dark gray.

We could in 24% of the citations confirm that they accurately referred to relevant primary literature. Of the 107 citations, 56 referred to relevant primary literature, whereof 30 were considered inaccurate and 26 were accurate. Inaccurate citations were found in 13 of 14 reviews (Figure 3). The inaccuracy was attributable to confusion with different or unclear definitions for tire wear (TWP, TRWP, or TWP+BWP), units (per tire or vehicle; milligram or microgram), and particle size (PM_10_ or PM_2.5_). Some reviews quoted factors that were not stated by the reference, quoted references that did not estimate tire emission factors, or made assumptions that contradicted the cited references. Ideally, all pie charts in Figure 3 are blank with green borders (not filled or semifilled).

Uncited references were examined in addition to the existing citations. In total, only 34% of the relevant primary studies that could have been quoted were quoted by the reviews. The 14 reviews combined did not make a total of 81 possible citations to relevant primary studies, although the primary literature was published before the reviews (referred to as “uncited”). Additionally, eight of the reviews made 30 citations to relevant primary studies without quoting the emission factors for unexplained reasons (referred to as “cited, but excluded”). The emission factors from 11 relevant primary studies were not quoted in any of the 14 reviews: Pierson and Brachaczek^31^ (1974), Allen et al.^50,51^ (2006, 2007), Gustafsson et al.^33^ (2009), Gehrig et al.^35^ (2010), Zhang et al.^46^ (2020), Beji et al.^34^ (2021), Hicks et al.^25^ (2021), Farahani et al.^40^ (2022), De Oliveira et al.^44^ (2024), Khardi^38^ (2024), and Fang et al.^29^ (2024). The mean emission factors for tire wear PM_10_ from “uncited” and “cited, but excluded” references were 1.6 and 1.1 mg/vkm, respectively. The corresponding medians were both relatively low at 0.2 mg/vkm. Note that all known types of methodologies are represented in the unquoted studies: indoor road simulators as well as real-world experiments such as on-road, roadside, and tunnel measurements.

**Figure 4:**
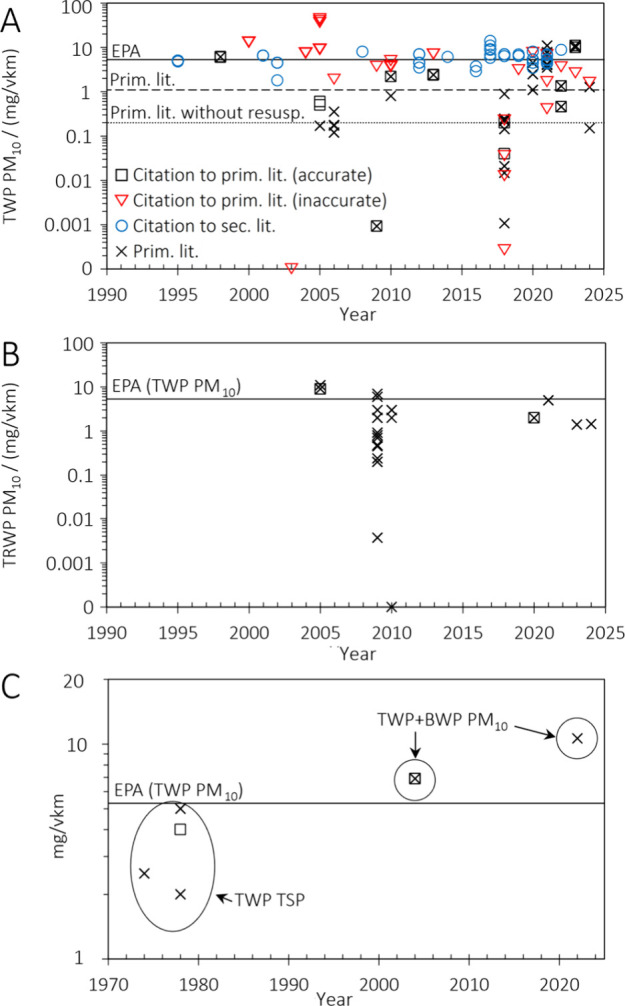
Emission factors for light-duty vehicles and mixed fleets with unstudded tires: (A) TWP PM_10_, (B) TRWP PM_10_, and (C) TWP TSP and TWP+BWP PM_10_. Accurate and inaccurate citations to primary literature, citations to secondary literature, and estimates from primary literature are shown as squares, triangles, circles, and crosses, respectively. The year refers to the year of publication from the primary study or the cited reference.

The numerous inaccurate citations prompted us to investigate whether they were random or systematic. Figure 4 illustrates a quantitative comparison of 130 emission factors from 14 reviews with 61 emission factors from 25 relevant primary studies. For comparison, TWP PM_10_ emission factors for light-duty vehicles from the EPA and medians from the primary literature including and excluding studies with resuspended TWP are shown as solid, dashed, and dotted horizontal lines, respectively.

Surprisingly, more than half of the emission factors within relevant primary literature, as defined by us, have been exclusively misquoted or not quoted in the reviews. In total, 47 triangles, 30 squares, 38 crosses without, and 23 crosses with corresponding squares were found (Figure 4). Ideally, every cross would be framed by a square, while no triangles would appear. Notably, the TWP PM_10_ mean of 8.5 mg/vkm for the triangles (inaccurately quoted primary studies) was higher than 32 of 35 emission factors reported by primary studies. The mean emission factor of 6.5 mg/vkm for tire wear PM_10_ according to the reviews decreases to 4.1 mg/vkm when excluding primary references that did not estimate mass-based tire wear PM_10_ emission factors and when replacing the mistaken figures with the corresponding measured figures. The mean further decreases to 2.5 mg/vkm when excluding secondary references. When including ‘cited, but excluded’ references, an emission factor of 1.7 mg/vkm is obtained, which falls between our mean tire wear PM_10_ emission factors of primary literature excluding (1.1 mg/vkm) and including (2.7 mg/vkm) studies with resuspended TWP. This analysis demonstrates three aspects: 1) misquotations and 2) frequent citations to other secondary literature have introduced systematically higher emission factors compared to the underlying primary sources, which 3) represented the upper end of primary literature. Note that the demonstration of aspects 1) and 2) is independent from our selection of primary studies, as it relies on the studies selected by the reviews. For aspect 3), the reviews did not clarify why sources with lower emission factors were systematically excluded, implying a selection bias.

**Figure 5:**
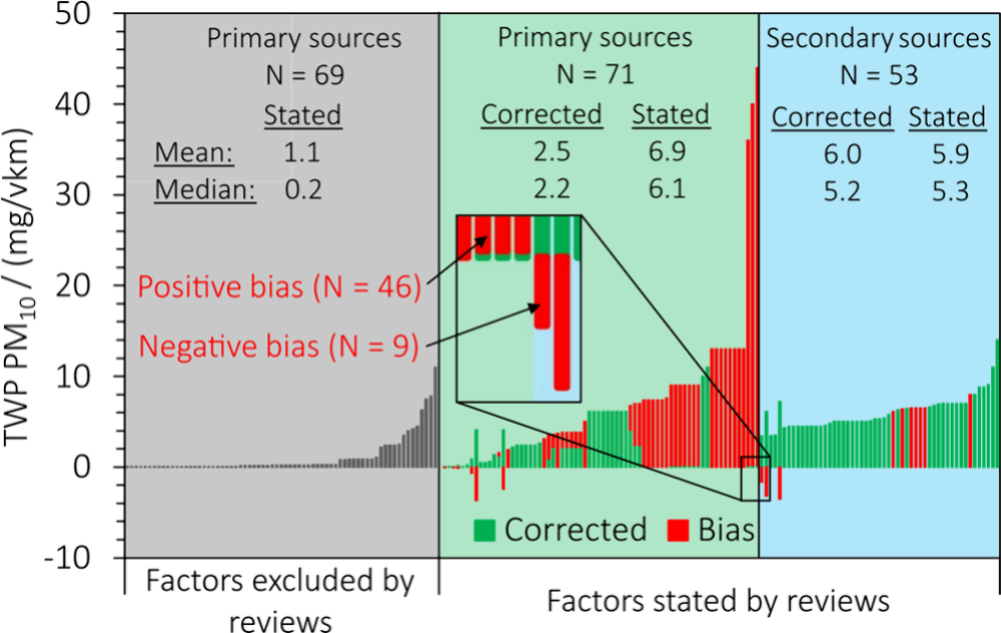
Histogram of emission factors for tire wear PM_10_ in the reviews referring to primary sources (green background) or other secondary sources (blue background). Some primary sources were found by the reviews but not quoted for unexplained reasons (gray background). Differences between reviews and the cited sources (“corrected”) are shown in red to visualize the bias introduced by misquotations.

The significance of the bias introduced by misquotations, frequent citations to other secondary sources, and exclusion of low emission factors is further illustrated in Figure 5, which visually demonstrates four aspects for tire wear PM_10_: 1) high emission factors in the reviews are more likely misquoted than low ones. Most extreme cases are the highest emission factors for tire wear PM_10_ around 40 mg/vkm from two reviews quoting Kupiainen et al.,^32^ although this reference measured 200 times lower emission factors of ∼0.17 mg/vkm. 2) The differences between the factors stated by the reviews and the cited sources are more likely to be positive than negative. In other words, misquotations systematically introduced a bias toward higher and not lower emission factors. 3) Although they are relatively accurate, the citations from the reviews to other secondary sources are 2–3 times higher than the corrected primary studies selected by the same reviews. 4) The majority of the ‘cited, but excluded’ emission factors are below 1 mg/vkm and therefore not in agreement with the other factors stated by the reviews, which may explain why these factors were not considered.

Tire wear PM_10_ emission factors exclusively below EPA’s factor for light-duty vehicles (5.3 mg/vkm) were reported by 13 of 16 primary studies, in 7 studies by 1 order of magnitude or more. Of 35 TWP PM_10_ emission factors based on on-road, roadside, tunnel, and road simulator experiments with different road surfaces (concrete, asphalt, sandpaper) and different driving styles, only 7 emission factors were found high enough to support tire wear PM_10_ emission factors of ≥5.0 mg/vkm, whereof 6 were estimated for mixed fleets including resuspended TWP. Additionally, only 6 of 26 factors for TRWP PM_10_, TWP+BWP PM_10_, and TWP TSP were higher than 5.0 mg/vkm despite the mass contributions from bigger or nontire particles (Figure 4B,C). This comparison between measured and propagated emission factors questions the validity of currently established emission factors as a best estimate for TWP PM_10_ from light-duty vehicles, including the factors of EPA^21,22^ with 5.3 mg/vkm and EEA^20^ with 6.4 mg/vkm.

### 3.3 Critical Analysis of Selected Examples

We elaborate on selected examples to demonstrate the discrepancy between primary and secondary research. For example, some citations referred to references that did not report tire emission factors (EMPA^76^) or no emission factors at all (Suleiman et al.,^77^ Keuken et al.^78^). Keuken et al. (2010) were potentially cited as an attempt to find Keuken et al.^71^ (1999), which may have been confused with a more recent study from the same lead author. It is unclear why numerous studies quoted EMPA at 13 mg/vkm for tire wear PM_10_, as we were unable to locate this figure in the report, whose final version is exclusively available in German language. Our perception has been confirmed by the lead author of that study, who emphasized by email that no estimates for tire emission factors have been made, as further stated by other authors^79^ of the same research institute. Nevertheless, 8 of 14 reviews quoted EMPA^76^ with an emission factor of 13 mg/vkm. Boulter^66^ (2005) indicated to have cited EMPA indirectly through Lükewille et al.^64^ (2001), which was the first publication quoting EMPA (2000) with 13 mg/vkm to our knowledge. The origin of this figure is unclear according to e-mail correspondence with the lead author of the EMPA report and one author of Lükewille et al.

Potentially, the 13 mg/vkm are based on the emission inventory from EPA in 1985, which stated a tire wear PM_10_ emission factor of 0.002 g/mile/vehicle (= 1.2 mg/vkm). In 1995, the EPA inaccurately quoted this factor as 0.002 mg/mile/tire (= 5 mg/vkm) for the new PART5 emissions model, presumably due to a mix-up of units (Text S4). Incorrect conversion from miles to kilometers (multiplying instead of dividing by 1.61) may have eventually led to 13 mg/vkm, as for example seen in the calculations from Alexandrova et al. in 2007.^51^ The citations to EPA with 5 mg/vkm and EMPA with 13 mg/vkm are of great relevance, as they form the rationale behind today’s tire wear PM_10_ emission factors from both EPA and EEA (Text S2). Note that both agencies use these PM_10_ emission factors to derive estimates for PM_2.5_ as well as for vehicles other than light-duty vehicles.

Kupiainen et al.^32^ estimated PM_10_ emission factors of ∼10 mg/vkm resulting from abrasion at the tire and road interface. The authors did not classify these particles as tire or road wear particles. However, 8 of 10 reviews presented these results as emission factors for TWP alone. It would be likely more accurate to interpret the reported emission factors as mainly road rather than tire particles, given that Kupiainen et al. quantified the mineral content in the PM_10_ fraction to ≥90% (by number). Their chemical mass balance implied average PM_10_ tire contributions of 1.5% with a maximum of 5%, which was accounted for in one^66^ of ten reviews.

Luhana et al.^41^ estimated an emission factor of 6.9 mg/vkm for TWP+BWP PM_10_, which was accurately quoted by 2 of 7 reviews. Surprisingly, the other 5 reviews quoted this study with 7.4 mg/vkm tire wear PM_10_. This can be explained as Luhana et al. additionally measured a total tire tread loss of 74 mg/vkm, which was used by Grigoratos and Martini^69^ to calculate emission factors for tire wear PM_10_ assuming an elsewhere found aerosolization efficiency of 10%. Notably, the asserted approach using aerosolization efficiency did not appear within Luhana et al., who quantified BWP and TWP combined to a lower emission factor. The other 4 reviews directly cited Luhana et al. with 7.4 mg/vkm for tire wear PM_10_, although this factor was calculated by Grigoratos and Martini.

Sjödin et al.^43^ estimated an emission factor of 2.2 mg/vkm using roadside measurements and a chemical mass balance (CMB). The authors used a source profile for TWP based on TRWP partly generated with studded tires, which introduced considerable uncertainty due to increased road wear, as highlighted by the authors. Surprisingly, 6 of 7 reviews quoted this reference with an emission factor of 3.6 or 3.8 mg/vkm. The origin of these figures is not discernible to us and two authors of Sjödin et al. according to email correspondence. Only one review quoted this study with 2.2 mg/vkm.^24^

### 3.4 Emergence and Perception of the Discrepancy

Although such efforts must be decoupled from the best measures of airborne TWP emissions, environmental agencies may have intentionally set high emission factors to use a conservative approach for human health protection or induce industry to demonstrate better performance. However, given the prevalence and nature of frequently misquoted emission factors, it seems more plausible that the discrepancy between the primary and secondary literature has arisen unintentionally. Note that most of the mistaken figures are associated with half-truths or terminological inconsistencies, emphasizing the unintentional character of the misquotations.

The three examples of EMPA,^76^ Luhana et al.,^41^ and Sjödin et al.^43^ demonstrate how 7 of 14 reviews reproduced emission factors from other secondary studies while citing primary research. This pseudodirect citation practice (referencing the primary study but taking the values from the secondary source) introduced a lack of traceability and prevented others from comprehending where the stated numbers originated. This practice likely emerges from an ambivalence between the challenge of encapsulating a wide research subject, the aversion toward indirect citations, and the temptation to trust reputable secondary sources. Language barriers and difficulties in accessing key publications further exacerbated the persistence of the discrepancy. The potential role of the illusory truth effect^80^ shall not be ignored. This phenomenon describes the increased probability to perceive frequently reiterated statements as being more truthful because of the repeated exposure.

No authors were found who considered a discrepancy between primary and secondary literature as a contributing reason for the variety of emission factors found in the literature, demonstrating the novelty of our work. Nevertheless, we have found a few authors who questioned the general opinion in the literature. De Oliveira et al.^44^ and Charbouillot et al.^36^ proposed that currently established emission factors for airborne tire wear are potentially overestimated. Mennekes and Nowack^79^ highlighted that country-based total TWP emission studies lack scientific support. Charbouillot et al., DEFRA,^75^ and Harrison et al.^81^ implied that emission factors in emission inventories are based on old studies. We are unaware of historical studies stating emission factors of ≥6.4 mg/vkm for tire wear PM_10_.

### 3.5 Implications of the Discrepancy

It may matter decisively whether emission factors for tire wear PM_10_ are, for example, 1 mg/vkm rather than 5 mg/vkm. The latter exceeds the legal limit for exhaust emissions in the European Union^18^ and United States,^19^ whereas the former remains below these limits (note differences in size and chemical composition between exhaust emissions and TWP). Research focus in academia and industry is determined by current emission factors. The higher the factors, the more motivation is created to conduct research and the more pressure is built up on governments to take measures. Resource management and political agendas should be based on emission factors, reflecting scientific evidence.

Currently established emission factors for airborne TWP are part of national emission inventories and have been widely used for governmental reports^75,82,83^ and academic research,^84−92^ for example to study the impact from electric vehicles on air pollution.^7,8,93−98^ The conclusions of these studies can be misleading if the underlying emission factors are inaccurate. For example, the increased vehicle mass associated with electric vehicles is potentially accompanied by less direct PM_10_ emissions than those currently anticipated due to the TWP bias.

Primary study authors may compare their results with the literature and conclude that more research is needed due to an alleged lack of consensus. They may not recognize the mistaken emission factors in the secondary literature. For example, Panko et al.^42^ contextualized their result (2.4 mg/vkm) by comparing it with a literature benchmark for light-duty vehicles ranging from 2 to 13 mg/vkm for tire wear PM_10_. Notably, the lower end of this benchmark was 2 times higher than the median emission factor we have found in the primary literature (including studies with resuspended TWP). Similarly, although the factors stated by Hicks et al.^25^ (∼6 mg/vkm) and Beddows et al.^26^ (∼10 mg/vkm) were the highest emission factors we have found in primary literature, the authors did not express astonishment after comparing their results with emission factors from EEA. On the other side, we found several authors in primary research questioning their results due to “relatively low” emissions in comparison to misquoted emission factors from secondary literature. Woo et al.^15^ commented on the discrepancy, stating: ‘this significant difference is presumed to be the reason why laboratory measurements cannot accurately reflect the wear characteristics that occur under real-world driving conditions’. Similar statements were made by other authors,^34,39,41,48,49,52^ where “relatively low” emission factors of airborne TWP were contextualized with a secondary literature benchmark. However, this benchmark contained bias, as revealed by our analysis. Authors stating to have measured “relatively high” emissions were not found.

Secondary literature appears to propagate high emission factors while excluding low factors, also due to concerns from primary study authors. These concerns, however, may result from unexpectedly low emissions relative to secondary literature: an example of circular reasoning driven by the illusory truth effect. We observed cases where primary literature authors quoted their own work with misperceived emission factors without being able to explain by email where they originated, demonstrating the dominance of frequently misquoted figures in comparison to the measured figures.

### 3.6 Limitations

Low emission factors for tire wear PM_10_ do not imply that TWP is unimportant for air quality as these factors only provide insights into mass metrics for directly emitted particles with aerodynamic diameters below 10 μm. Although TWP are believed to contribute significantly to bigger airborne particles,^99,100^ the environmental and health impacts of these bigger particles remain unclear. Additionally, the chemical composition or shape of tire wear PM_10_ may pose health risks despite potentially low exposures.

Mass-based PM_10_ emission factors do not accurately reflect tire wear nanoparticles and number-based emissions in general. The mass contribution from ultrafine tire particles to tire wear PM_10_ could be higher than is currently anticipated. De Oliveira et al.^44^ observed that the rubber mass within tire wear PM_10_ was most dominated by particles smaller than 0.39 μm. Tonegawa and Sasaki^47^ stated tire wear PM_2.5_/PM_10_ ratios close to unity. During harsh braking, Kim and Lee^52^ reported surprisingly high mass concentrations of nanoparticles along with nanoparticle growth due to coagulation and condensation of volatile tire material. Considering the hypothesized role of evaporation and condensation of an unidentified tire component,^15,49,52,53,101−104^ it seems plausible that tire wear nanoparticles are semivolatile oil droplets subject to coalescence. Note that Williams and Cadle^30^ in 1978 reported roughly equal mass emissions of gaseous tire wear and tire wear TSP.

Finally, the large mass of nonairborne emissions from tires along with unclear effects for the environment and health are concerning and subject of ongoing research. It is critical that studies carefully differentiate between direct and indirect emissions into the atmosphere. Tire particles may degrade, decrease in size, resuspend, and thus become PM_10_ indirectly.^105^ It is reported that the wear-related stress is accompanied by chemical alterations accelerating chemical and biological degradation of TWP, hypothetically due to cleavage of covalent bonds within the rubber framework.^30,106,107^ Williams and Cadle showed in 1978 that approximately 30% of styrene–butadiene-rubber in sedimentary TWP is unvulcanized–a fraction 15 to 30 times higher than in tire tread.^30^ The breakdown and subsequent evaporation of rubber potentially explains the elevated levels of zinc on the surface of TWP reported by Li et al.^107^ This evidence may shift the focus from directly to indirectly emitted airborne TWP, underlining the roles of microplastic emissions and physical removal of curbside dust. Consequently, the assumption of chemical similarities between tire wear particles and tire tread seems to be questionable, which may have relevant implications when quantifying tire wear using chemical tracers. The term TWP is potentially misleading when referring to airborne matter originating from tires, for example, if rubber and filler separate during degradation.

## 4 Future Perspectives

Currently accepted mass-based emission factors of airborne TWP do not adequately reflect the scientific evidence from primary research. Secondary literature reports tire wear PM_10_ emission factors 2 to 30 times higher than primary research, depending on the definition of primary research, the statistical metrics, and whether studies quantifying resuspended TWP are included or not. The discrepancy, dating back to 1995, is due to misunderstandings and misquotations that arose during the knowledge transfer from the primary to secondary literature. Inaccurate quotations have led to an unfounded but prevalent opinion of relatively high emission factors, which permeated emission inventories from environmental agencies and hindered the formation of a scientific consensus. Thus, revision of current tire wear PM_10_ emission factors and the associated conclusions is warranted. The accuracy of prospective knowledge transfers between primary and secondary research can be improved by using clear terminologies and avoiding pseudodirect citations.

## Supporting Information Available

Elaborations on methodologies, EPA’s and EEA’s emission factors, Rauterberg-Wulff’s dissertation, and the studies in the 1970s (PDF)
Overview, a detailed table of all analyzed emission factors, and summaries of the data used in Figure 3, 4, and 5 (XLSX)
