# Supplementary material for: Airborne Tire Wear Particles: A Critical Reanalysis of the Literature Reveals Emission Factors Lower than Expected
Source: Environ Sci Technol Lett. 2024 Nov 24;11(12):1296–307. doi: 10.1021/acs.estlett.4c00792 (PMC11636205; doi:10.1021/acs.estlett.4c00792)
Supplement: Supplementary file 1 — ez4c00792_si_001.pdf [file ez4c00792_si_001.pdf]

Supporting information for

Airborne Tire Wear Particles:

a Critical Reanalysis of the Literature Reveals Emission Factors Lower than Expected

Siriel Saladin[1,*], Adam Boies[2], and Chiara Giorio[1,*]

[1]Yusuf Hamied Department of Chemistry, University of Cambridge, Cambridge CB2 1EW, United Kingdom

[2]Department of Engineering, University of Cambridge, Cambridge CB2 1PZ, United Kingdom

*Corresponding authors:
Siriel Saladin: sls87@cam.ac.uk
Chiara Giorio: chiara.giorio@atm.ch.cam.ac.uk

Table of contents

S1. Methodologies to estimate emission factors

S1.1 Laboratory experiments

S1.2 Real-world experiments

S2. Emission factors from EPA and EEA

S3. Dissertation of Rauterberg-Wulff

S4. Studies in the 1970s and their perception

S1. Methodologies to estimate emission factors

Emission factors of airborne tire wear can be measured in different ways, which are subject to individual challenges, limitations, and uncertainties. We believe all individual approaches are subject to substantial uncertainties. However, the results of all approaches combined may provide insight, as the characteristics of the constraints differ between different approaches. With respect to the diversity of different methodologies, it seems unlikely that all reported emission factors are under- or overestimated. Nevertheless, methodological uncertainties should be considered when making a best estimate of emission factors for airborne TWP. It can be distinguished between laboratory and real-world experiments.

S1.1 Laboratory experiments

Laboratory studies rely on road simulators and can be classified into tire-on-drum,[1–6] tire-in-drum,[7–9] and tire-on-track[10–12] methodologies, whereas various surface materials are used. The main text of this study uses the term 'asphalt track' to refer to tire-on-track road simulators equipped with asphalt. Some authors use wear resistant surfaces such as sandpaper[4] (usually silicon carbide or aluminum oxide) or slip resistant tapes such as safety walk.[3,5] There appears to be a common opinion that PM contributions from sandpaper are negligible[3,4,7] – an assumption which, to our knowledge, has not been validated or invalidated. The community further seems to agree that sandpaper represents real-world abrasion in a highly uncertain fashion. We are not aware of compelling evidence to support the authenticity of the wear mechanism with sandpaper – neither are we aware of evidence to the contrary.

Some authors[10–12] used circular road simulators with asphalt pavement instead of sandpaper. These experiments tend to be accompanied by higher wear rates due to slip at the tire and road interface because of the circular track.[11] In contrast, high slip increases tire temperature which may directly or indirectly alter the abrasion mechanism, potentially reducing the emissions of airborne tire wear particles (TWP). Tires on road simulators were reported to become sticky, preventing the release of PM_{10}. In response, mineral third-body particles can be added to obtain more realistic conditions and to prevent the tire surface from becoming tacky.[8] The benefit of using asphalt or third-body particles comes with a downside when investigating TWP, given that the resulting particles will be a mixture of tire and road wear particles (TRWP). Although the proportions between tire and road wear are unknown, road simulators with asphalt may reveal a relatively accurate maximum for TWP emissions - if TRWP is X mg/vkm, then TWP alone cannot be higher than X mg/vkm under these conditions.

Particle collection and characterization can shed light on the tire contribution to TRWP. In this case, tracer compounds can be used. Ideally, these tracers are specific for tires and not present in bitumen

or other particle sources – an unfavorable requirement as tires and bitumen are both, at least partly, based on crude oil. Bitumen additives such as styrene-butadiene-styrene further complicate the differentiation from styrene-butadiene-rubber (SBR) in tires.[13] Most authors assume that the mass fraction of the selected tracer in bulk tire material is similar to tire wear particles. However, significantly different chemical compositions for tire wear and tire tread were reported.[1,14]

Despite the uncertainties, the methods with road simulators have a common advantage: the modelling part of the experiment is simplified as the experimental setup is often based on a semi-sealed cabinet with a well-defined volume and controllable background level. The particles can be extracted through a pipe and integrated over time to obtain total PM_{10} emissions. Alternatively, extraction can be switched off and emissions are measured inside the enclosure as they accumulate. In this case, the particle loss rate should be determined prior to a sample run as performed by Sjödin *et al.*[15]

S1.2 Real-world experiments

The second type of experiment is based on locations in real-world such as roadside,[16–21] on-road,[22–25] or road tunnels,[26–30] which have a major advantage compared to road simulators: the investigated tire wear is by nature representative for real-world tire wear. However, real-world approaches require particle collection followed by chemical analysis to quantify the contribution from TWP, as the collected particles were emitted by a mix of various sources such as tire, brake, and road wear as well as exhaust and non-road sources.

Some authors quantified tracer compounds like zinc,[19,20,28] SBR,[28] styrene,[22] or pyrolysis products[18,25] of rubber and then calculated total PM_{10} mass of TWP, although the specificity of these tracers are usually unclear. Other authors used chemical mass balances based on positive matrix factorization or previously estimated source profiles for selected emission sources.[15,16,21,26,27,30] In these cases, the implications of unknown specificities are eliminated, at the cost of new uncertainties related to other emission sources that are not or only inaccurately considered in the mass balance. Additionally, tracer compounds and source profiles are subject to uncertainties related to changing chemical compositions as discussed previously. The latter uncertainties can be avoided when using positive matrix factorization. However, how can a carbon-rich factor be attributed to tire wear if, for example, bitumen wear may be similarly plausible?

In contrast to road simulators and on-road study designs, scientists at the roadside and in tunnels do not know which tires the passing vehicles are equipped with. Consequently, the investigators need to analyze a multitude of various tires to determine an average chemical composition that they consider representative. During on-road campaigns, scientists can eliminate this uncertainty by using their own vehicle equipped with known tires and a mobile test facility. On the other hand, on-road experiments may collect an unknown fraction of TWP causing the calculated emission factor to be underestimated

to an unknown extent. These study designs require collection efficiencies to be quantified, which is challenging and inevitably introduces uncertainties that may overestimate or underestimate emission factors.

Real-world experiments face the challenge of resuspension. Were the measured particles emitted freshly or were they the result of previously emitted TWP that settled and resuspended due to the vehicle wake or the tire road interaction? The bias of resuspension can be reduced by equipping the test vehicle with additional sampling probes to perform background subtraction[22,24] or by intensive cleaning of the test track.[23] The latter strategy is only applicable for private test tracks and even then, it will not be realistic to remove all road dust prior to an experiment. The challenge of resuspension has been demonstrated by Sjödin *et al.*[15] and Gustafsson *et al.*[11] who thoroughly cleaned the asphalt track of an indoor road simulator using a pressure washer. Despite all efforts, the authors still observed indications for resuspension.

Lastly, it is generally more challenging in real-world than laboratory experiments to link the emitted mass of particles with the corresponding driven distance, for example during urban roadside experiments. The emission sources are mobile and they constantly change their location following roads in various directions resulting in a highly dynamic spatial distribution of airborne TWP, both in terms of time and location. Was the measured TWP emitted by vehicles from the road nearby or a road further away? It becomes unclear what traffic data to use for contextualization of the quantified TWP mass. A workaround is the methodology based on concentration differences between the sampling site and a background site (roadside increments).[19,20] Concentrations of traffic-related emissions such as NO_x and CO_2 are calculated using elsewhere reported emission factors and then divided by the measured NO_x and CO_2 increment to reveal dilution rates. This approach assumes that NO_x and CO_2 emission factors are accurate and that TWP dilute in a similar manner as gases. Note that these roadside measurements do not differentiate between freshly emitted and locally resuspended TWP. Alternatively, measurements in single-direction tunnels are subject to reduced dispersion and dilution of TWP, simplifying the quantification as well as the model. In addition, the TWP concentration at the beginning of a tunnel can be subtracted from the concentration at the end of the tunnel allowing an estimate for the mass of emitted particles within the tunnel. This strategy is expected to reduce the effects of resuspension, although unclear to what extent. In contrast, traffic in a tunnel tends to be characterized by less brake events and less tire wear, indicating that emission factors from tunnels may be lower compared to average driving conditions. For our work, estimates from real-world experiments were classified as 'directly emitted' if targeted measures were taken to separate resuspended from freshly emitted particles (extensively cleaning the test track, performing a local background subtraction, or making use of single-lane road tunnels). Consequently, estimates from other real-world studies were classified as 'resuspended and directly emitted'.

S2. Emission factors from EPA and EEA

The current MOVES4[31] model (released 2023) of the United States Environmental Protection Agency (EPA) states emission factors for directly emitted airborne TWP based on the predecessor model MOVES3[32] (released 2020), which is based on the MOVES2014[33] model (released 2014). The documentation from MOVES3 clarifies how the current emission factors were derived. The authors estimated an average tire weight loss and quoted Luhana *et al.*[27] with 'between around 1 percent and 15 percent by mass of passenger car tire wear material is emitted as PM_{10}'. EPA derived PM_{10} aerosolization efficiencies of 8 % (average of 1 % and 15 %) to be multiplied by the estimated tire weight loss to obtain the current tire wear PM_{10} emission factor. For the conversion from PM_{10} to $PM_{2.5}$, EPA cited Kupiainen *et al.*[10] who measured a $PM_{2.5}/PM_{10}$ ratio of 0.15. Note that Kupiainen *et al.* investigated TRWP (not TWP). Similarly, the aerosolization efficiencies of 1 % to 15 % stated by Luhana *et al.* were not experimental results. Instead, Luhana *et al.* performed a literature review for TWP citing Williams and Cadle[1] with 4 mg/vkm for TSP, Rauterberg-Wulff[26] with 6.1 mg/vkm for PM_{10}, EPA[34] in 1995 with 4.8 mg/vkm for PM_{10}, and EMPA[35] with 13 mg/vkm for PM_{10}. Note that the citation of EMPA was indirect through Lükewille *et al.*[36] Based on these citations, Luhana *et al.* considered tire wear PM_{10} emission factors of 4 to 6 mg/vkm to be representative. They divided the lower emission factor by the highest (360 mg/vkm) and the higher emission factor by the lowest (40 mg/vkm) elsewhere reported tire weight loss rate and thus obtained a minimum and maximum PM_{10} aerosolization efficiency of 1 % and 15 %, respectively. They divided the highest emission factor (13 mg/vkm) by 40 mg/vkm to obtain the most conservative aerosolization efficiency of 30 % which has been widely[32,33,37–39] quoted. EPA classifies road abrasion and resuspension as stationary sources and hence records them separately from mobile sources such as tire wear (see Text S4 for more details).

The portrayal of the four references behind the current emission factor from EPA appears questionable. Note that EMPA in the year 2000 did not state emission factors for tire wear particles, which was confirmed by email correspondence with the lead author of this study. Furthermore, the citation with 4.8 mg/vkm to EPA in 1995 is subject to misunderstood units (per tire instead of per vehicle), causing the cited emission factor to be 4 times higher than originally stated by EPA (see Text S4 for more details). Lastly, the quoted emission factor from Williams and Cadle refers to TSP and not PM_{10}, indicating that the dissertation of Rauterberg-Wulff is the only considered study which estimated emission factors for tire wear PM_{10}. However, the representativeness of Rauterberg-Wulff's study for other primary studies appears uncertain, given that its emission factor is the highest of 26 estimates we have found in primary literature for directly emitted tire wear PM_{10} (see Text S3 for more details).

We have not found an explanation in the guidebook for the rationale behind the EMEP/EEA tire wear PM_{10} emission factors. However, the appendix on a website[40] from the UNECE Task Force on Emission

Inventories and Projections (TFEIP) provides clarification. TFEIP supported the development of the guidebook in 2003. Interestingly, TFEIP quoted the same 4 references as Luhana *et al.* with identical emission factors. By comparing these emission factors with the tire weight losses for light-duty vehicles in literature, TFEIP concluded that between 1 % and 10 % of passenger car tire wear is emitted as PM_{10}. They multiplied an average tire tread loss of 64 mg/vkm with 10 % to obtain an emission factor for tire wear PM_{10} of 6.4 mg/vkm for the guidebook. The rationale behind the assumed tire wear $PM_{2.5}/PM_{10}$ ratio of 0.70 is unclear to us. It seems possible that the guidebook used the $PM_{2.5}/PM_{10}$ ratio of 0.70 from their literature review referring to Berdowski *et al.*[41] from TNO. We cannot verify this citation as TNO did not share the report because it was 'entirely outdated'. For the conversion from PM_{10} to TSP, the guidebook assumes a PM_{10}/TSP ratio of 0.6 as explained by TFEIP. Surprisingly, this ratio refers to Miguel *et al.*[42] who investigated resuspended dust from paved roads (not tire wear). The rationale for the tire wear $PM_{2.5}$, PM_{10}, or TSP emission factors within the EMEP/EEA guidebook has not been updated since implementation in 2003. Nevertheless, the EMEP/EEA tire wear emission factors have constantly been refined to account for different vehicles or speeds.

The similarities between the literature reviews from TFEIP,[40] Luhana *et al.*,[27] Boulter,[37] and the EMEP/EEA guidebook[43] are apparent yet not surprising considering that one author has contributed to all these publications. It is evident that the rationales for emission factors of airborne tire wear from the EMEP/EEA guidebook and EPA's MOVES2014, MOVES3, and MOVES4 models rely on the same cluster of references that emerged around the year 2003. The 23 relevant primary literature studies published after 2003 are not considered by EEA or EPA. Both environmental agencies did not update their factors which align surprisingly well with today's scientific reviews (see main text). The good agreement may be coincidental or indicative of a self-fulfilled prophecy.

S3. Dissertation of Rauterberg-Wulff

To the knowledge of us and Rauterberg-Wulff[26], this dissertation from 1998 was the first published study to estimate tire wear emission factors for PM_{10}. The author collected fine ($PM_{2.5}$) and coarse ($PM_{2.5–10}$) particles inside the tunnel Tegel in Berlin (Germany) and thermographically estimated the ratio of OC2 (organic carbon degrading between approximately 350 °C and 620 °C) to elemental carbon (EC). Rubber from tires thermally degrades within this range. A chemical mass balance was applied using the measured OC2/EC ratio of the coarse particles. Comparison with previously estimated OC2/EC ratios for diesel soot, brake pads, and tires enabled the quantification of EC from TWP in the coarse fraction using the formula $EC_{tire,\ coarse} = (OC2_{coarse} – 0.2 \times EC_{coarse}) / (1.5 – 0.2)$. An OC2/EC ratio of 0.2 was applied for diesel soot, resulting from the measured OC2/EC ratio of the fine fraction assuming all OC2 and EC originated from diesel soot. The same ratio was found for brakes after analyzing 21 brake pads. An OC2/EC ratio of 1.5 for tires was obtained by analyzing 18 tires. The resulting mass of tire elemental carbon ($EC_{tire,\ coarse}$) was converted to TWP using the measured EC mass fraction average of 28 % from 18 tires.

We highlight the pioneering work of Rauterberg-Wulff, while discussing the methodology from today's perspective. The 'Tunnelleitzentrale Berlin' (local tunnel control center) confirmed by telephone that asphalt pavement was present in the tunnel at the time of the experiments. Bitumen in asphalt consists of organic carbon degrading at approximately 400 °C.[44] The model of Rauterberg-Wulff assumed negligible carbon contributions from the road – an assumption which seems uncertain today. Bitumen is dominated by organic carbon, implying that it may alter the mass balance disproportionately in favor of tire wear. Although the emission factor for PM_{10} from road bitumen is poorly understood, evidence published after 1998 suggests that the bitumen mass in microplastics[45] and in TSP[46] is not significantly lower compared to TWP.

S4. Studies in the 1970s and their perception

To our knowledge, the two studies in the 1970s (Pierson and Brachaczek[28] and Williams and Cadle[1]) were the first and only ones to estimate emission factors for airborne TWP before 1998 (excluding studies where tire debris was collected directly behind the wheel without size cut-offs). It should be noted that both studies reported TSP and not PM_{10}. The term PM_{10} first appeared in literature around 1984, when it was proposed by EPA to replace the TSP standard.[47] The two studies used different methodologies (road tunnel and road simulator) and found similar emission factors of around 3 mg/vkm. Note that Pierson and Brachaczek additionally referred to mixed fleets while Williams and Cadle referred to light-duty vehicles only. The authors of both studies concluded that the contribution from TWP to airborne PM is minor or negligible. Pierson and Brachaczek measured tire TSP aerosolization efficiencies of no more than 2 % to 7 % in the Allegheny Tunnel, Pennsylvania. Furthermore, approximately 20 % of SBR in TSP was PM_{10} according to the measurement at Rotunda Drive, Michigan (Figure 7 from Pierson and Brachaczek). Combining both results, we calculate a maximum tire wear PM_{10} aerosolization efficiency of approximately 0.4 % to 1.75 % according to our interpretation of Pierson and Brachaczek's results. In comparison, EEA and EPA assume tire PM_{10} aerosolization efficiencies of 10 % and 8 %, respectively. Pierson and Brachaczek proposed that 'the important material resulting from tire wear may be abraded from the roadway rather than from the tire'. This hypothesis appears verified decades later for the PM_{10} fraction.[2,8,10,11,15,48,49] The conclusion of minor contributions from airborne tire wear seems reasonable at the time in view of the high exhaust emissions, but should be revised for today with respect to the significantly decreased overall PM concentrations and potentially different asphalt or tire mixtures. The emission factor of Pierson and Brachaczek was not quoted by the 14 reviews.

EPA in the compilation[50] of air pollutant emission factors from 1985 (AP-42, Volume II) stated tire wear emission factors of 0.002 g/mile/vehicle (1.2 mg/vkm) for PM_{10} and 0.0005 g/mile/vehicle (0.3 mg/vkm) for $PM_{2.5}$, citing both studies from the 1970s. EPA assumed that these emission factors are valid for all vehicle classes and speeds, which seems a reasonable assumption due to the scarce availability of data. To our knowledge, EPA 1985 is the first secondary literature stating emission factors for airborne TWP. It is unclear to us how EPA derived these emission factors, as both studies did not estimate emission factors for tire wear PM_{10} or $PM_{2.5}$. However, the size distribution of airborne SBR (Figure 7 from Pierson and Brachaczek) suggests that ~20 % (by mass) of airborne SBR is attributable to the PM_{10} fraction, prompting us to calculate emission factors of 0.5 mg/vkm for tire wear PM_{10} of mixed fleets and light-duty vehicles. The tire wear PM_{10} emission factor of 1.2 mg/vkm as proposed by EPA is 2.4 times higher and seems to be a conservative interpretation.

In 1995, a PART5 draft user's guide[34] from EPA quoted EPA 1985 with 0.002 g/mile/tire, which is four times higher with respect to passenger cars, appearing to be the result of a misunderstanding (per tire versus per vehicle). In 1995, EPA additionally published the fifth version of the AP-42 Volume I report,[51] which has often been put in context with tire emissions,[27,36,40,52–54] although it covers stationary emission sources only. Mobile emission sources such as tire wear are covered in Volume II. It is unclear whether a Volume II report from 1995 exists or whether it has already been superseded by the PART5 model from EPA, according to email correspondence with EPA.

EPA in the year 1995 is widely quoted with 4.8 or 5 mg/vkm for tire wear PM_{10},[27,36–38,40,52,53,55–57] obtained by multiplying the emission factor of 0.002 g/mile/tire by 4. TFEIP[40] provides clarification how EPA's factor was converted for the EMEP/EEA guidebook. Some of the TWP PM_{10} citations to EPA in 1995 refer to Volume I and Volume II, whereas other citations refer to only Volume I or the user's guide. In some reviews, EPA 1995 is cited in the main text without being listed in the bibliography. We assumed that all these citations referred to the PART5 model and thus we classified them as accurate. However, we can only speculate why the reviews exclusively quoted EPA from 1995 and not the underlying compilation of air pollutant emission factors from 1985. PART5 (implemented in 1995) was superseded by the MOBILE6.1 model (synonymous with MOBILE 6.2) in 2003, which also referred to EPA 1985 with 0.002 g/mile/tire instead of 0.002 g/mile/vehicle.[58]

References

(1) Williams, R. L.; Cadle, S. H. Characterization of Tire Emissions Using an Indoor Test Facility. *Rubber Chem. Technol.* **1978**, *51* (1), 7–25.

(2) Aatmeeyata; Kaul, D. S.; Sharma, M. Traffic Generated Non-Exhaust Particulate Emissions from Concrete Pavement: A Mass and Particle Size Study for Two-Wheelers and Small Cars. *Atmos. Environ.* **2009**, *43* (35), 5691–5697. https://doi.org/10.1016/j.atmosenv.2009.07.032.

(3) Park, I.; Kim, H.; Lee, S. Characteristics of Tire Wear Particles Generated in a Laboratory Simulation of Tire/Road Contact Conditions. *J. Aerosol Sci.* **2018**, *124*, 30–40. https://doi.org/10.1016/j.jaerosci.2018.07.005.

(4) Kim, G.; Lee, S. Characteristics of Tire Wear Particles Generated by a Tire Simulator under Various Driving Conditions. *Environ. Sci. Technol.* **2018**, *52* (21), 12153–12161. https://doi.org/10.1021/acs.est.8b03459.

(5) Woo, S.; Jang, H.; Mun, S.; Lim, Y.; Lee, S. Effect of Treadwear Grade on the Generation of Tire PM Emissions in Laboratory and Real-World Driving Conditions. *Sci. Total Environ.* **2022**, *838* (P4), 156548. https://doi.org/10.1016/j.scitotenv.2022.156548.

(6) Zhang, Q.; Fang, T.; Men, Z.; Wei, N.; Peng, J.; Du, T.; Zhang, X.; Ma, Y.; Wu, L.; Mao, H. Direct Measurement of Brake and Tire Wear Particles Based on Real-World Driving Conditions. *Sci. Total Environ.* **2024**, *906*, 167764. https://doi.org/10.1016/j.scitotenv.2023.167764.

(7) Foitzik, M.-J.; Unrau, H.-J.; Gauterin, F.; Dörnhöfer, J.; Koch, T. Investigation of Ultra Fine Particulate Matter Emission of Rubber Tires. *Wear* **2018**, *394–395*, 87–95. https://doi.org/10.1016/j.wear.2017.09.023.

(8) Schläfle, S.; Unrau, H. J.; Gauterin, F. Influence of Longitudinal and Lateral Forces on the Emission of Tire–Road Particulate Matter and Its Size Distribution. *Atmosphere (Basel).* **2023**, *14* (12), 1780. https://doi.org/10.3390/atmos14121780.

(9) Schläfle, S.; Unrau, H. J.; Gauterin, F. Influence of Load Condition, Tire Type, and Ambient Temperature on the Emission of Tire–Road Particulate Matter. *Atmosphere (Basel).* **2023**, *14* (7), 1095. https://doi.org/10.3390/atmos14071095.

(10) Kupiainen, K. J.; Tervahattu, H.; Räisänen, M.; Mäkelä, T.; Aurela, M.; Hillamo, R. Size and Composition of Airborne Particles from Pavement Wear, Tires, and Traction Sanding. *Environ. Sci. Technol.* **2005**, *39* (3), 699–706. https://doi.org/10.1021/es035419e.

(11) Gustafsson, M.; Blomqvist, G.; Brorström-Lundén, E.; Dahl, A.; Gudmundsson, A.; Hjort, M.; Johansson, C.; Jonsson, P.; Swietlicki, E. *Nanoparticles from the Abrasion of Tyres and Pavement (Translation of VTI Report 660)*; VTI, 2009.

(12) Alves, C. A.; Vicente, A. M. P.; Calvo, A. I.; Baumgardner, D.; Amato, F.; Querol, X.; Pio, C.; Gustafsson, M. Physical and Chemical Properties of Non-Exhaust Particles Generated from Wear between Pavements and Tyres. *Atmos. Environ.* **2020**, *224*, 117252. https://doi.org/10.1016/j.atmosenv.2019.117252.

(13) Rødland, E. S.; Samanipour, S.; Rauert, C.; Okoffo, E. D.; Reid, M. J.; Heier, L. S.; Lind, O. C.; Thomas, K. V.; Meland, S. A Novel Method for the Quantification of Tire and Polymer-Modified Bitumen Particles in Environmental Samples by Pyrolysis Gas Chromatography Mass Spectroscopy. *J. Hazard. Mater.* **2022**, *423* (Part A), 127092. https://doi.org/10.1016/j.jhazmat.2021.127092.

(14) Li, K.; Yu, J.; Kong, D.; Chen, X.; Peng, Y.; Wang, L. Differential Cytotoxicity to Human Cells in Vitro of Tire Wear Particles Emitted from Typical Road Friction Patterns: The Dominant Role of Environmental Persistent Free Radicals. *Chemosphere* **2023**, *343*, 140256. https://doi.org/10.1016/j.chemosphere.2023.140256.

(15) Sjödin, Å.; Ferm, M.; Björk, A.; Rahmberg, M.; Gudmundsson, A.; Swietlicki, E.; Johansson, C.; Gustafsson, M.; Blomqvist, G. *Wear Particles from Road Traffic - a Field, Laboratory and Modelling Study. Final Report*; IVL, 2010. https://doi.org/10.13140/RG.2.2.18594.35524.

(16) Abu-Allaban, M.; Gillies, J. A.; Gertler, A. W.; Clayton, R.; Proffitt, D. Tailpipe, Resuspended Road Dust, and Brake-Wear Emission Factors from on-Road Vehicles. *Atmos. Environ.* **2003**, *37* (37), 5283–5293. https://doi.org/10.1016/j.atmosenv.2003.05.005.

(17) Bukowiecki, N.; Lienemann, P.; Hill, M.; Furger, M.; Richard, A.; Amato, F.; Prévôt, A. S. H.; Baltensperger, U.; Buchmann, B.; Gehrig, R. PM10 Emission Factors for Non-Exhaust Particles Generated by Road Traffic in an Urban Street Canyon and along a Freeway in Switzerland. *Atmos. Environ.* **2010**, *44* (19), 2330–2340. https://doi.org/10.1016/j.atmosenv.2010.03.039.

(18) Panko, J. M.; Chu, J.; Kreider, M. L.; Unice, K. M. Measurement of Airborne Concentrations of Tire and Road Wear Particles in Urban and Rural Areas of France, Japan, and the United States. *Atmos. Environ.* **2013**, *72*, 192–199. https://doi.org/10.1016/j.atmosenv.2013.01.040.

(19) Beddows, D. C. S.; Harrison, R. M.; Gonet, T.; Maher, B. A.; Odling, N. Measurement of Road Traffic Brake and Tyre Dust Emissions Using Both Particle Composition and Size Distribution Data. *Environ. Pollut.* **2023**, *331* (P1), 121830. https://doi.org/10.1016/j.envpol.2023.121830.

(20) Hicks, W.; Beevers, S.; Tremper, A. H.; Stewart, G.; Priestman, M.; Kelly, F. J.; Lanoisellé, M.; Lowry, D.; Green, D. C. Quantification of Non-Exhaust Particulate Matter Traffic Emissions and the Impact of COVID-19 Lockdown at London Marylebone Road. *Atmosphere (Basel).* **2021**, *12* (2), 190. https://doi.org/10.3390/atmos12020190.

(21) Farahani, V. J.; Altuwayjiri, A.; Taghvaee, S.; Sioutas, C. Tailpipe and Nontailpipe Emission Factors and Source Contributions of PM10 on Major Freeways in the Los Angeles Basin. *Environ. Sci. Technol.* **2022**, *56* (11), 7029–7039. https://doi.org/10.1021/acs.est.1c06954.

(22) Tonegawa, Y.; Sasaki, S. Development of Tire-Wear Particle Emission Measurements for Passenger Vehicles. *Emiss. Control Sci. Technol.* **2021**, *7*, 56–62. https://doi.org/10.1007/s40825-020-00181-z.

(23) Charbouillot, T.; Janet, D. C.; Schaal, P.; Beynier, I.; Boulat, J. M.; Grandchamp, A.; Biesse, F. Methodology for the Direct Measurement of Tire Emission Factors. *Sci. Total Environ.* **2023**, *863*, 160853. https://doi.org/10.1016/j.scitotenv.2022.160853.

(24) Beji, A.; Deboudt, K.; Khardi, S.; Muresan, B.; Lumière, L. Determinants of Rear-of-Wheel and Tire-Road Wear Particle Emissions by Light-Duty Vehicles Using on-Road and Test Track Experiments. *Atmos. Pollut. Res.* **2021**, *12* (3), 278–291. https://doi.org/10.1016/j.apr.2020.12.014.

(25) De Oliveira, T.; Muresan, B.; Ricordel, S.; Lumière, L.; Truong, X. T.; Poirier, L.; Gasperi, J. Realistic Assessment of Tire and Road Wear Particle Emissions and Their Influencing Factors on Different Types of Roads. *J. Hazard. Mater.* **2024**, *465*, 133301. https://doi.org/10.1016/j.jhazmat.2023.133301.

(26) Rauterberg-Wulff, A. Beitrag Des Reifen- Und Bremsenabriebs Zur Rußimmission an Straßen, Ph.D. Thesis, Technische Universität Berlin, 1998.

(27) Luhana, L.; Sokhi, R.; Warner, L.; Mao, H.; Boulter, P.; McCrae, I.; Wright, J.; Osborn, D. *Measurement of Non-Exhaust Particulate Matter*; 2004.

(28) Pierson, W. R.; Brachaczek, W. W. Airborne Particulate Debris from Rubber Tires. *Rubber Chem. Technol.* **1974**, *47* (5), 1275–1299.

(29) Allen, J. O.; Alexandrova, O.; Kaloush, K. E. *Tire Wear Emissions for Asphalt Rubber and Portland Cement Concrete Pavement Surfaces*; Arizona State University, 2006.

(30) Zhang, J.; Peng, J.; Song, C.; Ma, C.; Men, Z.; Wu, J.; Wu, L.; Wang, T.; Zhang, X.; Tao, S.; Gao, S.; Hopke, P. K.; Mao, H. Vehicular Non-Exhaust Particulate Emissions in Chinese Megacities: Source Profiles, Real-World Emission Factors, and Inventories. *Environ. Pollut.* **2020**, *266* (P2), 115268. https://doi.org/10.1016/j.envpol.2020.115268.

(31) EPA. *Overview of EPA's Motor Vehicle Emission Simulator (MOVES4)*; 2023.

(32) EPA. *Brake and Tire Wear Emissions from Onroad Vehicles in MOVES3*; 2020.

(33) EPA. *Brake and Tire Wear Emissions in MOVES2014*; 2014.

(34) EPA. *Draft User's Guide to PART5: A Program for Calculating Particle Emissions from Motor Vehicles*; 1995.

(35) Eidgenössische Materialprüfungs- und Forschungsanstalt (EMPA). *Beitrag Des Strassenverkehrs Zu Den PM10- Und PM2.5-Immissionen. Schlussbericht*; NFP41 Verkehr und Umwelt, 2000.

(36) Lükewille, A.; Bertok, I.; Amann, M.; Cofala, J.; Gyarfas, F.; Heyes, C.; Karvosenoja, N.; Klimont, Z.; Schoepp, W. *A Framework to Estimate the Potential and Costs for the Control of Fine Particulate Emissions in Europe*; IIASA, 2001.

(37) Boulter, P. G. *A Review of Emission Factors and Models for Road Vehicle Non-Exhaust Particulate Matter*; TRL Limited, 2005.

(38) Grigoratos, T.; Martini, G. *Non-Exhaust Traffic Related Emissions. Brake and Tyre Wear PM*; Joint Research Centre, 2014. https://doi.org/10.2790/21481.

(39) Jan Kole, P.; Löhr, A. J.; Van Belleghem, F. G. A. J.; Ragas, A. M. J. Wear and Tear of Tyres: A Stealthy Source of Microplastics in the Environment. *Int. J. Environ. Res. Public Health* **2017**, *14* (10), 1265. https://doi.org/10.3390/ijerph14101265.

(40) Task Force on Emission Inventories and Projections (TFEIP). *Automobile tyre and brake wear*. https://www.eng.auth.gr/mech0/lat/PM10/ (accessed 2024-06-13).

(41) Berdowski, J. J. M.; Mulder, V. C.; Vesschedijk, Ä. J. H.; Zandveld, P. Y. J. *Particulate Matter Emissions (PM10 - PM2.5 - PM0.1) in Europe in 1990 and 1993*; TNO, 1997.

(42) Miguel, A. G.; Cass, G. R.; Glovsky, M. M.; Weiss, J. Allergens in Paved Road Dust and Airborne Particles. *Environ. Sci. Technol.* **1999**, *33* (23), 4159–4168. https://doi.org/10.1021/es9904890.

(43) EEA. *EMEP/EEA Air Pollutant Emission Inventory Guidebook 2023*; 2023.

(44) Jiménez-Mateos, J. M.; Quintero, L. C.; Rial, C. Characterization of Petroleum Bitumens and Their Fractions by Thermogravimetric Analysis and Differential Scanning Calorimetry. *Fuel* **1996**, *75* (15), 1691–1700. https://doi.org/10.1016/S0016-2361(96)00169-X.

(45) Järlskog, I.; Strömvall, A. M.; Magnusson, K.; Gustafsson, M.; Polukarova, M.; Galfi, H.; Aronsson, M.; Andersson-Sköld, Y. Occurrence of Tire and Bitumen Wear Microplastics on Urban Streets and in Sweepsand and Washwater. *Sci. Total Environ.* **2020**, *729*, 138950. https://doi.org/10.1016/j.scitotenv.2020.138950.

(46) Fauser, P.; Tjell, J. C.; Mosbaek, H.; Pilegaard, K. Tire-Tread and Bitumen Particle Concentrations in Aerosol and Soil Samples. *Pet. Sci. Technol.* **2002**, *20* (1–2), 127–141. https://doi.org/10.1081/LFT-120002092.

(47) EPA. *Proposed Revisions to the National Ambient Air Quality Standards for Particulate Matter*; 1984.

(48) McAtee, B.; Gustafsson, M.; Blomqvist, G.; Gudmundsson, A.; Sweet, L.; Panko, J.; Finley, B. Physio-Chemical Analysis of Airborne Tire Wear Particles. *Toxicol. Lett.* **2009**, *189*, S205. https://doi.org/10.1016/j.toxlet.2009.06.621.

(49) Gustafsson, M.; Blomqvist, G.; Gudmundsson, A.; Dahl, A.; Swietlicki, E.; Bohgard, M.; Lindbom, J.; Ljungman, A. Properties and Toxicological Effects of Particles from the Interaction between Tyres, Road Pavement and Winter Traction Material. *Sci. Total Environ.* **2008**, *393* (2–3), 226–240. https://doi.org/10.1016/j.scitotenv.2007.12.030.

(50) EPA. *AP-42: Compilation of Air Pollutant Emission Factors (Fourth Edition, Volume II)*; 1985.

(51) EEA. *AP-42: Compilation of Air Pollutant Emission Factors (Fifth Edition, Volume I)*; 1995.

(52) Klimont, Z.; Cofala, J.; Bertok, I.; Amann, M.; Heyes, C.; Gyarfas, F. *Modelling Particulate Emissions in Europe: A Framework to Estimate Reduction Potential and Control Costs*; IIASA, 2002.

(53) Baensch-Baltruschat, B.; Kocher, B.; Stock, F.; Reifferscheid, G. Tyre and Road Wear Particles (TRWP) - A Review of Generation, Properties, Emissions, Human Health Risk, Ecotoxicity, and Fate in the Environment. *Sci. Total Environ.* **2020**, *733*, 137823. https://doi.org/10.1016/j.scitotenv.2020.137823.

(54) EEA. *Air Pollutant Emission Inventory Guidebook 2007*; 2007.

(55) Panko, J.; Kreider, M.; Unice, K. *Chapter 7 - Review of Tire Wear Emissions: A Review of Tire Emission Measurement Studies: Identification of Gaps and Future Needs*; Elsevier Inc., 2018. https://doi.org/10.1016/B978-0-12-811770-5.00007-8.

(56) Liu, Y.; Chen, H.; Gao, J.; Dave, K.; Chen, J. Gap Analysis and Future Needs of Tyre Wear Particles. *SAE Tech. Pap.* **2021**, 2021-01–0621. https://doi.org/10.4271/2021-01-0621.

(57) Guo, D.; Wei, H.; Guo, Y.; Wang, C.; Yin, Z. Non-Exhaust Particulate Matter Emission from Vehicles: A Review. *E3S Web Conf.* **2021**, *268*, 01015. https://doi.org/10.1051/e3sconf/202126801015.

(58) EPA. *MOBILE6.1 Particulate Emission Factor Model Technical Description Final Report*; 2003.
